# Are Orthopaedic Trauma Surgeons Being Adequately Compensated for Treating Nonunions of the Femoral Shaft?: An Analysis of Relative Value Units

**DOI:** 10.5435/JAAOSGlobal-D-20-00163

**Published:** 2020-10-01

**Authors:** Azeem Tariq Malik, Carmen E. Quatman, Laura S. Phieffer, Safdar N. Khan, Thuan V. Ly

**Affiliations:** From the Department of Orthopaedics, The Ohio State University Wexner Medical Center, Columbus, OH.

## Abstract

**Introduction:**

We evaluated differences in reimbursement rates between native femoral shaft fractures treated with an intramedullary nail versus those undergoing repair of nonunion of femoral shaft fractures.

**Methods:**

The 2016 to 2017 American College of Surgeons—National Surgical Quality Improvement Program database was queried using International Classification of Diseases 10th Edition diagnosis codes and *Current Procedural Terminology* codes to identify patients undergoing surgery for native femoral shaft fractures and/or repair of nonunion of femoral shaft fracture with/without grafts.

**Results:**

The mean total relative value unit (RVU) and surgical time for each group were as follows: (1) native (RVU = 19.70, surgical time = 97.4 minutes), (2) nonunion w/out graft (RVU = 17.23, surgical time = 135.8 minutes), (3) nonunion w/graft (RVU = 18.88, surgical time = 164.5 minutes). Reimbursement rates decreased notably as complexity of case grew (native = $8.74/min versus nonunion w/graft = $6.07/min versus nonunion w/graft = $5.27/min; *P* < 0.001). The average reimbursement/case was $707 for native femoral shaft fracture, $618 for repair of nonunion w/out graft, and $678 for repair of nonunion with bone graft.

**Discussion:**

The study highlights the need for a change in the RVUs assigned to nonunions of the femoral shaft to ensure that the value of physician intensity is retained in future RVU evaluations.

Relative value units (RVUs) are a key component that serves as the foundation for medical reimbursement in our fee-for-service healthcare model.^1,2,3^ Each *Current Procedural Terminology* (CPT) code associated with the provision of medical service (eg, office visit, surgical procedure, or occupational/physical therapy etc.) is linked with a predefined number of RVUs that are multiplied by a conversion factor to determine reimbursements. The RVU associated with any CPT code consists of three major components—Work RVU, Practice Expenses RVU, and Malpractice RVUs (https://www.ama-assn.org/about/rvs-update-committee-ruc/rbrvs-overview).^4^ Contributing the largest component of the total RVU (nearly 50% of reimbursements associated with a CPT code), Work RVUs are reflective of physician intensity required to complete a certain medical task/procedure. In case of surgeries, Work RVUs are dependent on three major factors: (1) time required to complete the case, (2) amount of technical skill required, and (3) the stress/effort that a physician has to go through/use to do the surgery. Based on input/concerns raised by hospital administrators and physicians, the RVUs of selected procedures are often subjected to an annual audit by the American Medical Association RVU Update Committee to ensure that providers are being adequately compensated for the type/level of medical/surgical care they provided.

Despite these annual reviews, providers still criticize the RVU system of not being entirely reflective of surgical case complexity. In the orthopaedic realm, recent researches have shown that providers are being reimbursed at a lower rate ($/min) for performing a revision total joint arthroplasty (TJA) compared with a primary TJA despite the higher complexities and longer surgical times required in the former.^5^ A similar criticism, regarding the under-valuation of physician intensity required to treat complex fracture nonunions has been routinely voiced by orthopaedic trauma surgeons. With a lack of data to support the latter statement/concern, we conducted a national surgical database analysis to assess whether surgeons performing complex repairs of femoral shaft nonunions are being adequately compensated compared with intramedullary nailing for native femoral shaft fractures.

## Methods

### Database

This was a retrospective cohort study performed using the American College of Surgeons—National Surgical Quality Improvement Program (ACS-NSQIP) database.^6^ The ACS-NSQIP data set is a comprehensive surgical outcomes research repository that contains data from over 500 participating hospitals across the United States. With a strict audit and review process, the database is known to have over 98% accuracy and has been routinely used by orthopaedic trauma surgeons to study outcomes over the past decade. Starting from third quarter of 2015, ACS-NSQIP databases started including International Classification of Diseases 10th (ICD-10) diagnosis codes, in lieu of the older ICD-9 codes, to identify indication of procedure/surgery. Although the older ICD-9 coded lacked clinical granularity regarding the type of fracture, the newer ICD-10 codes can be used by researchers to identify nonunions for specific fracture types.

### Patient Selection

The 2016 to 2017 ACS-NSQIP database files were queried using CPT codes, and ICD-10 diagnosis codes to identify patients undergoing surgery for native femoral shaft fractures (CPT-27506) and/or repair of nonunion of femoral shaft fractures with/without graft (CPT-27470, CPT-27472). A detailed description of ICD-10 codes used to identify native and nonunion cases can be found in the Appendix (http://links.lww.com/JG9/A86). Patients undergoing secondary adjunct bone biopsies and/or concurrent surgery for proximal/distal femur, tibia, and/or upper extremity were removed to capture an isolated cohort of native and nonunion femoral shaft fractures only. The variable “WORKRVU” was combined with the RVUs of secondary adjunct procedural RVUs (eg, superficial implant removal; CPT-20670, CPT-11982) to calculate the total RVU of the procedure. It is important to mention that RVUs for concurrent coded removal of deep implants (CPT-20680) were not added to calculate the total RVU. This is because the CPT code for the primary nonunion procedure (27470 and 27472) is intended to incorporate/include/bundle the work done by the surgeon to remove deep implants, and insurance companies do not reimburse surgeons for the additional codes for deep implant removal.

### Statistical Analysis

Mean RVU per minute for each procedure type (native/27506 versus repair w/o graft/27470 versus repair w/graft/27472) was calculated. Reimbursement rate ($/min) was derived by multiplying the RVU per minute by a CMS-defined rate/conversion factor of $35.8887/RVU (https://www.cms.gov/Medicare/Medicare-Fee-for-Service-Payment/SustainableGRatesConFact). Average reimbursement/case was calculated by multiplying the reimbursement rate ($/min) by the total surgical time.

Kruskal-Wallis tests were used for carrying out statistical comparisons between the three groups to compare differences in mean total RVU, mean surgical time, RVU per minute, reimbursement rate, and average reimbursement/case. All statistical analyses were performed using SPSSv24 (IBM, Armonk, NY). Significance was set at *P* < 0.05.

## Results

A total of 425 patients were included in the final cohort—of which 358 (84.2%) underwent intramedullary nailing for a native femoral shaft fracture, 33 (7.8%) underwent repair of nonunion of femoral shaft fracture without use of graft, and 34 (8.0%) underwent repair of nonunion of femoral shaft fracture with use of a bone graft. The mean total RVU and surgical time for each group were as follows: (1) native (RVU = 19.70, surgical time = 97.4 minutes), (2) nonunion w/out graft (RVU = 17.23, surgical time = 135.8 minutes), (3) nonunion w/graft (RVU = 18.88, surgical time = 164.5 minutes). Reimbursement rates decreased notably as complexity of case grew (native = $8.74/min versus nonunion w/graft = $6.07/min versus nonunion w/graft = $5.27/min; *P* < 0.001). The average reimbursement/case was $707 for native femoral shaft fracture, $618 for repair of femoral shaft non-union w/out graft, and $678 for repair of nonunion of femoral shaft with bone graft (Table 1).

**Table 1:** Analysis of Reimbursement Rates and Average Reimbursements Between the Three Procedure Types

Variable		Native Intramedullary Nail of Femoral Shaft Fx (CPT-27506)	Repair Non-union Femoral Shaft Fx w/o Graft (Exchange Nail, CPT-27470)	Repair Non-union Femoral Shaft Fx With Bone Graft (CPT-27472)	*P*
N (%)		358 (84.2)	33 (7.8)	34 (8.0)	—
Mean total RVU		19.70 ± 0.60	17.23 ± 0.36	18.88 ± 0.51	<0.001
Mean surgical time (min)		97.4 ± 44.7	135.8 ± 80.9	164.5 ± 80.1	<0.001
Mean RVU/min		0.244 ± 0.106	0.169 ± 0.102	0.147 ± 0.090	<0.001
Mean reimbursement rate/min ($)		8.74 ± 3.80	6.07 ± 3.65	5.27 ± 3.22	<0.001
Average reimbursement/case ($)		707.1 ± 21.4	618.4 ± 12.9	677.5 ± 18.4	<0.001

CPT = *Current Procedural Terminology*, RVU = relative value unit

## Discussion

In the era of declining physician reimbursements and a push toward driving value rather than volume in care, our findings hold notable importance in the current changing healthcare landscape. Despite the notable complexity, higher effort and greater surgical times required for operating on nonunions, orthopaedic surgeons get reimbursed at a lower rate ($/min) for treating femoral shaft nonunions compared with intramedullary nailing of native femoral shaft fractures. Based on the current assigned RVUs, physician reimbursement per femoral shaft nonunion case can be $30 to $90 lower than intramedullary nailing for femoral shaft fracture despite the fact that nonunion cases can take, on average, an additional hour to complete.

Although evidence on appropriateness of RVUs in orthopaedic trauma remains an under-studied topic, past literature has highlighted major discrepancies in assigned RVUs, and subsequent physician reimbursement/compensation, in revision TJAs. Sodhi et al^5^ analyzed more than 107,000 individuals undergoing primary and revision total hip arthroplasties (THAs) and concluded that despite the higher complexity of revision cases; providers were being reimbursed at a lower rate for performing a revision THA ($8.93) compared with a primary THA ($9.33). In a similar analysis looking at total knee arthroplasties (TKAs), the authors found that revision TKAs were being under-valued with a physician reimbursement rate of $7.90/min compared with $9.33/min for primary TKAs.^7^ Malik et al^8^ analyzed over 6,000 cases of single- and double-component revision TKAs, and de-lineated that despite the longer surgical times and greater effort required for doing a double-component revision TKA providers are reimbursed at lower rate of $8.00/min compared with $9.58/min for a single-component revision TKA.

From an administrative point of view, our findings hold major importance, particularly for institutions that routinely take care of nonunions and are looking to launch “Non-union service lines.” Although these nonunion service lines might increase the quality in the delivery of care, they may have notable financial drawbacks. Given that nonunion cases may take up a notable proportion of a surgeon's surgical day, the financial compensation should at least be appropriately defined to ensure that case complexity, compared with native fractures, is taken into account. The lower RVUs associated with nonunion cases may also implicate certain hospital systems, where salary is based on the number of RVUs a surgeon can “pull in” for the institution.

From our perspective, few solutions exist to these problems. First, orthopaedic trauma surgeons need to advocate with their respective reimbursement committees to reconsider the bundling/coupling of deep implant removals with the CPT codes for nonunion cases. Conversely, an increase in the RVUs for nonunion cases can also ensure adequate reimbursements for these complex surgeries. Although some providers do use the “-22 modifier” to ensure higher reimbursement for complex cases, often times insurance companies do not approve the added RVUs associated with this modifier because of poor communication and/or incomplete documentation. Institutions should strongly ensure that when a “-22 modifier” is coded, the documentation is complete and up to date, which will ease the approval process by insurance companies.

A few limitations exist to the study. First, it is important to recognize that we only evaluated the physician intensity component of a total RVU (eg, WorkRVU) and did not look at facility expenses or malpractice components that may also have implications in subsequent health-policy changes. Furthermore, we did not have data on postacute care (office visits) that also play a role in determination of WorkRVU. However, given that nonunion fracture patients often require a more intensive postacute care protocol, compared with native fractures, the lower assigned RVUs further highlight the under-valuation of femoral nonunion surgeries. We also did not compare 30-day outcomes between the three procedure types because that was beyond the scope and/or objective of the study. Finally, the NSQIP database does not report CPT modifiers in their database, preventing us from identifying case instances where a -22 modifier was used and approved by the insurance company.

In conclusion, orthopaedic surgeons are reimbursed at a lower rate ($/min) for femoral shaft nonunions compared with intramedullary nailing for native femoral shaft fractures despite the higher complexity, greater effort, and longer surgical times required in the former. The study highlights the need for a change in the RVUs assigned to nonunions of the femur to ensure surgeons and being adequately compensated for treating a more complex and technical case.
